# Editorial: The role of evidence-based medicine and value-based medicine in clinical practice to enhance mental health

**DOI:** 10.3389/fpubh.2024.1487305

**Published:** 2024-09-16

**Authors:** Myriam M. Altamirano-Bustamante, Nelly F. Altamirano-Bustamante

**Affiliations:** ^1^Unidad de Investigación en Enfermedades Metabólicas, Centro Médico Siglo XXI, Instituto Mexicano del Seguro Social, Mexico City, Mexico; ^2^Servicio de Endocrinología, Instituto Nacional de Pediatría, Mexico City, Mexico

**Keywords:** mental health, axiology, evidence based medicine (EBM), values based approach, bioethics

Excellence in medicine demands an organizational culture founded on two principal pillars: evidence-based medicine (EBM) and values-based medicine (VBM) (1–4). This binomial EBM-VBM must be reinforced across all facets of clinical practice to ensure comprehensive and multidimensional patient care (5).

In recent years, patient mental health has increasingly become a challenge across all medical specialties, extending beyond the realms of psychology and psychiatry (6).

This Research Topic delves deeply into the EBM-VBM binomial and its pivotal role in integrating both approaches, ensuring that the pathophysiology of disease, its diagnosis, and treatment are pertinent to healthcare professionals, alongside a real-time bioethics framework that honors the ends and flourishment of the patients (7).

It is essential to strengthen the relationship between healthcare providers and patients to achieve comprehensive and multidimensional care that includes bio-psycho-social and economic aspects. This fosters communication, therapeutic adherence, trust, shared expectations, and support throughout the entire health-illness process. Enhancing patient mental health involves treating them as a person, respecting their intrinsic dignity, and supporting their integral flourishing (4).

Within this EBM-VBM framework, the article by Serrano-Zamago and Altamirano-Bustamante gains particular significance due to its focus on the ethical dimension of clinical practice. It is crucial to understand practices as a dynamic system comprising agents with common capacities and objectives, operating within a medical environment, whose actions are grounded in beliefs, theories, values, and virtues (8). In clinical practice, tacit ethical knowledge (TEK) is vital. This knowledge pertains to practical experiences involving values that are learned and transmitted implicitly, guiding actions without explicit awareness [Serrano-Zamago and Altamirano-Bustamante; (9–11)]. Currently, the lack of leaders dedicated to the integral wellbeing of patients has blurred the TEK, underscoring the urgent need to restore the sense of human dignity, respecting and promoting the integral flourishing of individuals regardless of their health status (5, 12–14).

TEK and its axiological dimension are examined in relation to clinical practice, with proposed educational strategies to enhance medical decision-making and address ethical dilemmas (15). Mental health, particularly in extreme conditions such as the health-illness process, which became evident during the pandemic, requires immediate attention to prevent the collapse of mental health clinics (16, 17).

In this context, Ujitoko et al.'s article highlights the importance of physical contact for survival, social bonding, and overall health. A glance, a handshake, or a smile may constitute the initial treatment offered by healthcare professionals. The COVID-19 pandemic exacerbated the depersonalization of clinical practice, making the rehumanization of healthcare urgent.

Kang et al. emphasize narrative medicine and the use of Chinese herbal medicine to enhance mental health. The articles by Juárez-Villegas et al. and Lu et al. demonstrate how mental health care is imperative across various specialties, revealing the intensification of syndromes such as depression, burnout, and anxiety in pediatric oncology and cardiology. They also highlight how ancient practices like acupuncture and VBM are crucial for integral treatment, with Juárez-Villegas et al. outlining best practices for the EBM-VBM binomial in end-of-life decision-making.

Finally, Attwood proposes educational strategies through art for teaching bioethics in clinical practice, presenting a revolutionary approach to identifying and discernment ethical dilemmas.

The promotion and strengthening of the EBM-VBM binomial through the axiological understanding of clinical practice is an evolving field and represents a significant opportunity to humanize medicine. This approach will foster therapeutic alliances with patients, improve therapeutic adherence, reduce comorbidities, hospital days, and emergency visits. The practical wisdom of medicine and its ethical dimension are essential for providing both quality and compassionate care to patients.
